# Probiotic and Bioactive Compounds in Foods: From Antioxidant Properties to Gut Microbiota Modulation

**DOI:** 10.3390/molecules31020345

**Published:** 2026-01-19

**Authors:** Berta Gonçalves, Alice Vilela, Alfredo Aires, Ivo Oliveira, Carla Gonçalves, Teresa Pinto, Fernanda Cosme

**Affiliations:** 1 Centre for the Research and Technology of Agro-Environmental and Biological Sciences (CITAB), Institute for Innovation, Capacity Building and Sustainability of Agri-Food Production (Inov4Agro), University of Trás-of-Montes e Alto Douro, Quinta de Prados, 5000-801 Vila Real, Portugal; bertag@utad.pt (B.G.); alfredoa@utad.pt (A.A.); ivo.vaz.oliveira@utad.pt (I.O.); carlagoncalves@utad.pt (C.G.); tpinto@utad.pt (T.P.); 2 Chemistry Research Centre-Vila Real (CQ-VR), University of Trás-of-Montes e Alto Douro, Quinta de Prados, 5000-801 Vila Real, Portugal; avimoura@utad.pt

**Keywords:** bioactive compounds, probiotics, plant-based foods, fermented foods, microbial metabolites, gut microbiota

## Abstract

Dietary bioactive compounds derived from plant-based and fermented foods act as plei-otropic modulators of human health, exerting antioxidant, anti-inflammatory, cardiopro-tective, neuroprotective, and metabolic effects beyond basic nutrition. Whole foods (fruits, vegetables, grains, nuts) provide synergistic mixtures of bioactives, whereas fermented foods generate a wide range of microbial-derived metabolites (peptides, organic acids) as well as probiotics that enhance nutrient bioavailability and support gut health. The gut microbiota plays a central mediating role in the biological effects of dietary bioactives through a dynamic, bidirectional interaction: dietary compounds shape microbial composition by promoting beneficial taxa and suppressing pathogens, while microbial metabolism converts these compounds into bioactive metabolites, including short-chain fatty acids, that profoundly influence host health. Despite their demonstrated health potential, the clinical translation of many dietary bioactives is limited by low bioavailability, which is influenced by digestion processes, food matrix and processing conditions, host genetics, and individual microbiota profile. Overcoming these limitations requires a deeper understanding of the synergistic interactions among dietary bioactives, probiotics, microbial metabolites, and host signaling pathways. This review provides an integrated perspective of the sources, mechanisms of action, and health effects of food-derived bioactive compounds and probiotic mediated effects, while highlighting current translational challenges and future directions for the development of effective functional foods and personalized nutrition strategies.

## 1. Introduction

Bioactive compounds derived from plant-based foods and fermented foods are increasingly recognized as pleiotropic modulators of human health, exerting biological effects beyond basic nutrition that contribute to disease prevention and overall well-being. Fruits, vegetables, whole grains, legumes, herbs, and fermented foods provide complex and diverse mixtures of bioactives, including polyphenols, flavonoids, phenolic acids, carotenoids, glucosinolates, alkaloids, vitamins, and probiotics, that act synergistically to influence multiple molecular and metabolic pathways [1,2,3,4,5]. These compounds modulate oxidative stress and inflammation, regulate gene expression, and alter gut microbiota composition, thereby supporting metabolic and immune homeostasis [2,6,7,8,9]. A comprehensive understanding of these interconnected mechanisms is essential for informing dietary recommendations and guiding the design of functional foods with targeted health-promoting properties.

The concept of functional foods has evolved considerably over the years. According to regulatory and scientific bodies such as the European Food Safety Authority (EFSA), the World Health Organization (WHO), and the U.S. Food and Drug Administration (FDA), functional foods can be either naturally occurring or deliberately modified to enhance their health-promoting properties [10]. More recently, Temple [11] defined functional foods as novel foods formulated to contain substances or live microorganisms that may provide health-enhancing or disease-preventing value, at concentrations that are both safe and sufficiently high to achieve the intended benefit. These added components may include nutrients, dietary fiber, phytochemicals, other bioactive substances, or probiotics. This concept includes foods that naturally contain bioactive compounds and foods that are intentionally formulated or processed to enhance health outcomes. While plant-based foods supply a wide array of chemical bioactives, such as polyphenols and carotenoids, fermented foods contribute complementary functional components through the activities of beneficial microorganisms and their metabolites [12,13,14]. 

During fermentation, microorganisms such as lactic acid bacteria and yeasts produce bioactive metabolites, including peptides, organic acids, vitamins, and viable probiotic cells, that support gut health, immune function, and metabolic regulation. Consequently, fermented foods, such as yogurt, kefir, kimchi, and tempeh, are widely recognized as functional foods because they contain bioactive ingredients that confer additional physiological benefits when consumed as part of a regular diet [15,16,17].

Increasing the consumption of plant-based and fermented foods naturally enhances dietary intake of bioactive compounds. Importantly, these foods typically provide complex mixtures of bioactives rather than isolated molecules, allowing synergistic interactions that may amplify health benefits [18,19]. Fruits and vegetables are major sources of dietary bioactive compounds: with berries providing phenolics and anthocyanins [20], leafy greens supplying carotenoids [21], cruciferous vegetables containing glucosinolates and isothiocyanates [22], and citrus fruits being rich in vitamin C and flavanones [23]. Whole grains, nuts, and seeds further contribute polyphenols, lignans, phytosterols, carotenoids, and omega-3 fatty acids, supporting antioxidant, anti-inflammatory, cardioprotective, and neuroprotective functions [24,25,26,27,28,29,30,31,32,33]. Although the bioavailability of many bioactives is influenced by digestion, food processing, host genetics, and gut microbiota composition, their health effects are often maximized when consumed within whole-food matrices, where synergistic interactions enhance stability, absorption, and efficacy. Collectively, higher consumption of fruits, vegetables, whole grains, nuts, and seeds is consistently associated with reduced risks of chronic diseases, underscoring the importance of these synergistic food sources in health promotion and functional food development [34,35].

Lactic acid bacteria and yeasts play a central role in the production of metabolites and probiotics that support gut health, immune modulation, nutrient absorption, and chronic disease prevention through microbiome-mediated mechanisms. In parallel, dietary bioactives influence gut microbiota composition by promoting beneficial bacteria such as *Lactobacillus* and *Bifidobacterium* while inhibiting pathogenic species. Moreover, microbial fermentation of dietary fibers leads to the production of short-chain fatty acids that further enhance gut integrity and immune function [36,37,38,39].

In addition to bioactives, fermentable oligo-, di-, and monosaccharides and polyols (FODMAPs) modulate the intestinal microbiota and may have both beneficial and adverse effects depending on individual tolerance [40,41].

**Figure 1:**
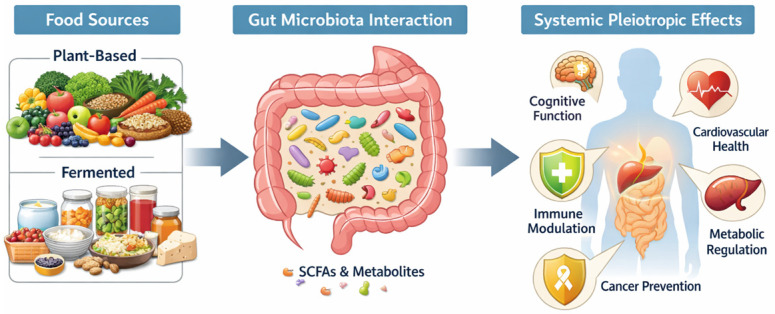
Schematic overview of the impact of dietary food sources on host health mediated by the gut microbiota, (Short-chain fatty acids—SCFAs).

The bidirectional interactions between dietary bioactive compounds and the gut microbiota represent a central yet unresolved theme in disease prevention research. While bioactives shape microbial composition, metabolism, and inflammatory responses, microbial transformation of these compounds generates metabolites that critically influence host signaling pathways and health outcomes. These interconnected processes remain incompletely understood, particularly within the context of complex food matrixes. Elucidating the synergistic relationships among dietary bioactives, microbial metabolites, and host signaling pathways is therefore crucial for advancing personalized nutrition and functional food development. This review synthesizes current evidence on the sources, mechanisms, and health effects of food-derived bioactive compounds, with a specific focus on their dynamic interactions with the gut microbiota in chronic disease prevention. It also highlights key translational challenges, including bioavailability and standardization, and outlines future directions for optimizing their application in preventive strategies (Figure 1).

## 2. Fruits and Vegetables Rich in Bioactives

Fruits and vegetables are well-recognized dietary sources of a wide array of bioactive compounds [1,2,3,4,5]. To date, more than 25,000 phytonutrients have been identified in plant-based foods [3]. Among the most common phytochemicals are polyphenols, flavonoids, isoflavones, carotenoids, phenolic acids, stilbenoids, coumarins, indoles, lignans, catechins, organosulfur compounds, anthraquinones, procyanidins, isothiocyanates, ginsenosides, saponins, and phenylpropanoids. Table 1 and Table 2 present representative examples of fruits and vegetables that serve as sources of these bioactive compounds.

Fruits and vegetables are key dietary sources of bioactive compounds. Berries, including blueberries, strawberries, and raspberries, are particularly rich in phenolic compounds and anthocyanins [20]. Leafy green vegetables such as spinach and kale provide high levels of carotenoids, notably lutein and zeaxanthin [21]. Cruciferous vegetables, including broccoli, cauliflower, and kale, are characterized by their high content of glucosinolates and isothiocyanates [22]. Citrus fruits, such as oranges, lemons, and grapefruits, are abundant in vitamin C and flavanones [23]. Average concentrations of selected bioactive compounds in different vegetable and fruit species are presented in Table 3.

From a dietary perspective, higher fruit and vegetable consumption increases the intake of bioactive compounds present as complex mixtures of phytochemicals, micronutrients, and dietary fiber that act synergistically to promote health [59,60]. When fruits and vegetables are consumed in their whole-food form, multiple components, such as dietary fibers, lipids, other bioactives, and micronutrients, interact in ways that can enhance absorption (for example, dietary fat facilitating carotenoid uptake), modulate metabolism, or delay degradation [26]. These interactions often result in cumulative or synergistic effects, ultimately increasing the overall bioefficacy of the food matrix compared with isolated compounds.

**Table 3:** Average content of the most representative and critical bioactive compounds in several vegetable and fruit species.

Food Specie	Bioactive Compounds	Average Content
Vegetables		
Spinach	Lutein + Zeaxanthin	12.2 mg/100 g
Broccoli	Glucoraphanin	0.2–0.3 mg/100 g
Kale	Kaempferol	1.0–1.5 mg/100 g
Cabbage	Sinigrin	0.5–1.0 mg/100 g
Carrot	Beta-carotene	8.0–10.0 mg/100 g
Collard greens	Quercetin	0.5–1.0 mg/100 g
Onion	Quercetin	0–91.0 mg/100 g
Tomato	Lycopene	19.41 mg/100 g
Fruits		
Apple	Quercetin	0.13–3.32 mg/100 g
Blueberry	Anthocyanins	6.0–8.0 mg/100 g
Cherry	Quercetin + Kaempferol	5.14–17.4 mg/100 g
Strawberry	Ellagic Acid	2.0–3.0 mg/100 g
Raspberry	Ellagic Acid	2.5–3.5 mg/100 g
Blackberry	Anthocyanins	5.0–7.0 mg/100 g
Goji Berry	Zeaxanthin	0.5–1.0 mg/100 g
Orange	Vitamin C	53.2 mg/100 g
Lemon	Vitamin C	53.0 mg/100 g
Grapefruit	Vitamin C	31.2 mg/100 g
Kiwi	Vitamin C	92.7–161.3 mg/100 g
Lime	Vitamin C	29.0 mg/100 g

Note: Values are approximate averages and may vary depending on factors such as cultivar, growing conditions, and preparation methods. Source data are derived from references [61,62,63,64,65].

Although bioactive compounds in fruits and vegetables are essential for human health, their physiological effectiveness is constrained by several factors. Many bioactives, such as polyphenols in berries or carotenoids in carrots and tomatoes, exhibit low bioavailability in their native forms and often require enzymatic or microbial transformation in the gut to become bioavailable [24]. In addition, complex biochemical changes occurring during the postharvest period can enhance the concentration and activity of certain compounds [66]. For example, postharvest ripening has been shown to increase proanthocyanidin levels by up to 76% in fruits from plum (*Prunus domestica* L.) cultivars [67]. Similarly, Carmona et al. [68] reported that anthocyanin content can be enhanced through post-harvest cold storage. Juice from cured Moro and Sanguinelli Polidori blood oranges contained 191.4 ± 1.4 mg/L of anthocyanins, compared with 85.7 ± 3.3 mg/L in fruits subjected to cold storage alone.

The stability of bioactive compounds can also be compromised by cooking, storage, or food processing. For instance, vitamin C in citrus fruits is highly susceptible to heat-induced degradation and prolonged storage [69]. Moreover, inter-individual factors, including genetic background, gut microbiota composition, age, and overall health status, further influence the absorption, metabolism, and tissue distribution of these compounds [25]. Finally, many protective effects, such as antioxidant and anti-inflammatory actions, require sustained and regular intake over time to achieve meaningful physiological benefits [70]. Collectively, these factors make it challenging to translate dietary intake into predictable health outcomes.

The health benefits of bioactive compounds in fruits and vegetables extend beyond disease prevention to supporting overall physiological resilience. Flavonoids and carotenoids, for example, have been shown to enhance cognitive function and slow age-related decline in older adults, particularly when five or more servings of fruits and vegetables are consumed daily [71]. Epidemiological evidence also indicates that higher intake of polyphenols, especially anthocyanins, catechins, and flavonols, is associated with a reduced risk of cardiovascular disease. In the NutriNet-Santé cohort in France (84,158 participants), individuals in the highest tertile of anthocyanin intake had approximately a 34% lower risk of cardiovascular disease compared to those in the lowest tertile (hazard ratio ≈ 0.66) [72].

Similarly, a systematic review and meta-analysis of 27 observational studies (cohort and case–control) [73] reported that high consumption of fruits and vegetables, particularly cruciferous, dark green, yellow, or orange vegetables, was associated with a reduced risk of endometrial cancer, with pooled relative risks ranging from approximately 0.64 to 0.81 across subgroups. Another recent analysis [74] demonstrated a nonlinear inverse dose–response relationship between cruciferous vegetable intake and colorectal cancer risk, with protective effects observed at intakes ≥20 g/day and optimal benefits at 40–60 g/day.

A broader systematic review of meta-analyses entitled “Associations of Fruit and Vegetable Intakes with Burden of Diseases” [75] confirmed that each 100 g/day increase in vegetable consumption was associated with reduced risk of coronary heart disease, all-cause mortality, and certain cancers, including renal cell carcinoma. However, benefits plateaued beyond approximately 300 g/day. This highlights the importance of a diverse, whole-food-based diet in delivering the physiological benefits of dietary bioactives, while reinforcing public health recommendations that promote achievable, evidence-based increases in fruit and vegetable consumption and inform the development of functional foods with targeted health-promoting properties.

## 3. Whole Grains, Nuts, and Seeds Rich in Bioactives

Whole grains, nuts, and seeds are distinguished by their high content of fiber-bound polyphenols (e.g., ferulic acid), lignans, phytosterols, tocols (α-, β-, γ-, and δ-tocopherols and tocotrienols), carotenoids, saponins, omega-3 fatty acids, along with prebiotic fibers such as β-glucans and arabinoxylans [27,28,29,32,76] (Table 4). These bioactive compounds are particularly associated with cardiometabolic health through antioxidant and anti-inflammatory actions, regulation of lipid metabolism and glucose homeostasis, and modulation of the gut microbiota, with additional neuroprotective benefits [27,30,31,77].

Whole grains, nuts, and seeds contribute distinct but complementary bioactive profiles that collectively support cardiometabolic health. Whole grains such as oats, barley, and wheat are rich in soluble fibers, particularly β-glucans and arabinoxylans, which play a key role in lowering cholesterol levels and improving glycemic control [78,79,80]. Nuts provide abundant unsaturated fatty acids, phytosterols, polyphenols, and tocopherols, that collectively reduce cardiovascular risk, improve endothelial function, and limit oxidative stress [81,82,83]. Seeds are particularly valued for their lignan, tocopherol, and alpha-linolenic acid content, which contribute to lipid regulation, anti-inflammatory effects, and hormone-related health benefits [84,85,86].

From a dietary perspective, these foods are consumed as complex matrices rather than isolated bioactive compounds, combining fibers, lipids, proteins, and phytochemicals. This structural complexity facilitates synergistic interactions that enhance the absorption, stability, and biological efficacy of bioactives [87,88]. In particular, the intrinsic lipid content of nuts and seeds plays a key role in promoting the intestinal absorption of lipid-soluble compounds, such as carotenoids [33] and tocols [34]. In parallel, the coexistence of dietary fiber and polyphenols in whole grains modulates gut microbiota composition, promoting the production of short-chain fatty acids and other microbial metabolites that contribute to systemic health benefits [66].

Epidemiological studies further underscore the protective role of these foods. Regular nut consumption is consistently associated with a lower risk of type 2 diabetes, cardiovascular disease, and all-cause mortality [35,89]. At the same time, higher whole-grain intake is consistently linked to reduced incidence of colorectal cancer and coronary heart disease [34,90]. Seeds, though typically consumed in smaller amounts, provide concentrated sources of lignans and omega-3 fatty acids with demonstrated benefits for cardiovascular and hormonal health [91]. Collectively, these findings position whole grains, nuts, and seeds as multifunctional dietary components that deliver complementary bioactives through complex food matrices, underscoring their relevance for targeted functional food design and reinforcing their central role within the framework of diet-based health promotion discussed in this review.

**Table 4:** Examples of bioactive compounds largely present in whole grains, nuts, and seeds.

Class of Bioactive/Phytochemical	Representative Examples	Key Foods	References
Phenolic acids	Chlorogenic acid, syringic acid, ferulic acid, and gallic acid	Wheat	[92]
Phenolic acids	*p*-hydroxybenzoic acid, caffeic acid, syringic acid, vanillic acid, and *p*-coumaric acid	Oat	[92]
Phenolic acids	*p*-coumaric acid, *o*-coumaric acid, and gallic acid	Corn	[92]
Flavonoids	isorhamnetin-3-*O*-rutinoside and isorhamnetin-3-*O*-glucoside (in combination), catechin, kaempferol-3-*O*-rutinoside, epicatechin, quercetin-3-*O*-galactoside, and isorhamnetin-3-*O*-galactoside	Almond	[93]
Lignans	Secoisolariciresinol, secoisolariciresinol diglucoside, matairesinol	Flaxseed	[94]
Phytosterols	β-sitosterol, campesterol, ergosterol, and stigmasterol	Nuts (almond, Brazil nut, cashew, hazelnut, macadamia, peanut, pecan, pine nut, pistachio, and walnut)	[95]
Tocols (Vitamin E forms)	α- and γ-Tocopherol, Tocotrienols	Sunflower seed, hazelnut, almond, wheat germ, barley	[96,97]
Omega-3 fatty acids	Alpha-linolenic acid (ALA)	Walnut, flaxseed, chia seed	[98,99]
Carotenoids	Lutein, Zeaxanthin, β-Carotene	Maize, pumpkin seed	[100,101]
Dietary fibers (prebiotics)	β-Glucans, Arabinoxylans	Oat, barley, wheat	[78,102]
Saponins	Soyasaponins, Avenacosides	Soybean, oat	[103,104]

## 4. Fermented Foods as a Source of Probiotic and Bioactive Metabolites

Fermented foods confer health benefits primarily through two complementary mechanisms. First, probiotics, defined as live microorganisms like lactic acid bacteria and yeasts, can temporarily colonize the gut, modulate the gut microbiome, enhance gut barrier function, and competitively inhibit pathogenic microorganisms. These effects depend on microbial viability and dosage, as well as on the protective role of the food matrix during digestion [105,106]. Second, fermentation produces bioactive metabolites, including peptides, short-chain fatty acids, polyphenols, organic acids, vitamins, and bacteriocins.

**Figure 2:**
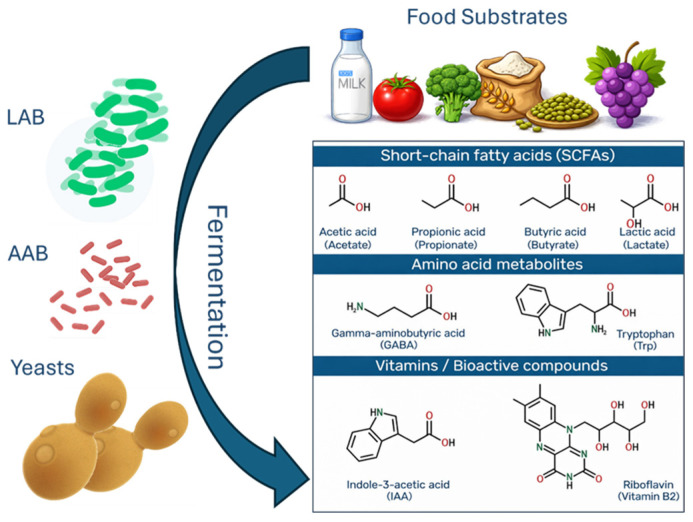
A schematic diagram illustrating the bioactive compounds generated through the fermentation processes of various food substrates by lactic acid bacteria (LAB), acetic acid bacteria (AAB), and yeasts.

The type and concentration of these compounds depend on the food matrix, microbial strains, and fermentation conditions. Lactic acid bacteria and yeasts, such as *Saccharomyces cerevisiae* and *Kluyveromyces marxianus*, are key contributors, producing bioactive peptides with antihypertensive, antioxidant, antimicrobial, and anti-inflammatory properties [107,108,109,110,111,112,113,114,115] (Figure 2).

Bioactive metabolites derived from fermented foods have been associated with improved gut health, enhanced immune function, reduced risk of chronic diseases (such as hypertension, diabetes, and obesity), and increased nutrient bioavailability. These benefits arise from the direct biological activity of fermentation-derived metabolites, their ability to modulate gut microbiota composition, and their interactions with host metabolic and immune signaling pathways [108,110,112,113,114,115,116,117,118].

Moreover, the diversity and functionality of bioactive metabolites produced during fermentation are strongly influenced by microbial community composition and environmental conditions. Metagenomic studies have shown that different fermentation matrices (such as dairy or plant-based substrates), harbor distinct biosynthetic gene clusters, resulting in unique metabolite profiles with variable biological activities [119,120,121]. These findings highlight the importance of microbial diversity and fermentation conditions in shaping the health-promoting potential of fermented foods.

### 4.1. Microbial Communities and Metabolism Across Fermented Foods

Microbial communities in fermented foods are highly diverse and dynamic, varying according to the substrate, geographical region, and fermentation method. Lactic acid bacteria, *Bacillus* spp., yeasts, and molds are commonly dominant; however, their relative abundance shifts throughout the fermentation process and differs between plant- and animal-based products [122,123,124,125,126,127,128,129,130]. Plant-based fermentations often harbor more heterogeneous lactic acid bacteria communities, whereas animal-based fermentations tend to display narrower microbial diversity [123,128,129,130].

Microbial metabolism during fermentation produces a wide array of metabolites, including amino acids, organic acids, B vitamins (such as riboflavin (B2), pyridoxine (B6), and cobalamin (B12)), vitamin C (ascorbic acid), polyphenols, and volatile flavor compounds. These metabolites enhance sensory quality, nutritional value, and preservation, and may also confer probiotic, antioxidant, and antimicrobial benefits [122,124,125,126,127,130,131,132,133,134]. Their biosynthesis is closely linked to microbial succession and the metabolic activity of specific taxa, with certain strains contributing disproportionately to the production of health-promoting or flavor-defining molecules [122,123,124,130,131,132,133,135,136].

Compounds generated during fermentation, such as organic acids, alcohols, esters, amino acids, and other metabolites, also influence microbial growth and succession, thereby shaping both the microbial community and the characteristics of the final product. The fermentation substrate (e.g., plant, dairy, or grain matrix) and its chemical composition largely determine which microorganisms dominate. For example, lactic acid bacteria dominate in environments rich in simple sugars, producing lactic acid that lowers pH and favors acid-tolerant species [66,105,106,127]. Furthermore, microbial metabolism produces compounds that selectively inhibit or promote specific taxa, thereby further modulating community structure [30,122,133,137].

Environmental factors such as pH, salt concentration, and temperature, as well as ingredient variations (e.g., addition of seafood to kimchi), can shift microbial succession, promote or suppress particular species, and modify metabolite profiles [106,122,138]. Multi-omics and correlation analyses have demonstrated strong associations between specific metabolites and microbial taxa (e.g., esters with *Saccharomyces* spp., acetic acid with *Acetobacter* spp.) [30,122,139]. By manipulating chemical inputs or environmental conditions, it is possible to intentionally steer microbial communities and flavor profiles during fermentation [106,133,138].

The microbial diversity and associated bioactivities across diverse fermented foods are summarized in Table 5.

Table 5 shows that microbial diversity in fermented foods is shaped by factors such as substrate type (plant, dairy, cereal, legume), geographical origin, and fermentation practices, resulting in distinct bioactive profiles across different food categories [128,129,131,145]. Lactic acid bacteria, for instance, dominate most fermented foods and play a central role in health-promoting activities, including probiotic effects, vitamin biosynthesis, and the production of antimicrobial compounds [123,131,140,142,143,147].

Plant-based and cereal-based fermentations, such as wine, vinegar, and kombucha, often exhibit higher microbial diversity and broader bioactive potential than more standardized dairy fermentations, reflecting differences in raw materials, processing conditions, and microbial succession dynamics [128,131,135,136,137,138,139,140].

### 4.2. The Case Study of Wine and Wine Vinegar

Microbial fermentation plays a fundamental role in the formation and enhancement of bioactive compounds in wine and vinegar. Yeasts, acetic acid bacteria, and lactic acid bacteria each contribute unique metabolites with health-promoting, sensory, and preservative properties. The strategic use of defined microbial consortia and starter cultures can further optimize these benefits, making wine and vinegar not only culinary staples but also functional foods with potential therapeutic value.

Acetic acid bacteria (e.g., *Acetobacter aceti*) are crucial in vinegar production, where they oxidize ethanol to acetic acid and may preserve or even increase the concentration of bioactive compounds like polyphenols, flavonoids, and unique statin-like molecules (e.g., brutieridin, melitidin in citrus vinegar) [148,149,150,151,152]. In addition, extracellular polymeric substances produced by acetic acid bacteria have attracted considerable interest in the food industry. These microbial polysaccharides enhance rheological properties, improve texture, and extend shelf life in bakery products [153,154]. They also serve as fat substitutes, stabilizers, and emulsifiers, including in edible starch-based films for food packaging [155,156]. Beyond technological applications, bacterial exopolysaccharides exhibit multiple health-related properties, including antitumor, antimicrobial, anti-inflammatory, hypocholesterolemic, antidiabetic, and immunostimulatory activities [135].

In wine, the probiotic potential of yeasts has received increasing attention, as reflected by the growing number of PubMed-indexed publications between 2016 and 2025. A search for “probiotic + yeast + wine” identifies the earliest relevant publication by Fleet in 2007 [157]. More recent studies (2025) have focused on non-traditional fermented beverages, such as lychee- and rice-based fermented beverages, highlighting their effects on intestinal function and sensory properties [158,159]. Expanding the search to “probiotic + yeast + grape + wine” retrieves a single study, “Antimicrobial Compounds in Wine” by Todorov et al. [160]. This study explores how terroir, climate, grape variety, and interactions between yeast and lactic acid bacteria influence wine maturation, storage, and sensory properties. This work also highlights antibacterial compounds derived from grapes and fermentation, including flavonoids, phenolic acids, and other bioactive metabolites that contribute to microbiological safety and the health-promoting properties [160].

Yeasts, especially *Saccharomyces* species, enhance the production of tryptophan-derived indole metabolites, such as serotonin and 3-indoleacetic acid, during wine fermentation. These levels can be further increased by using mixed or sequential yeast cultures [161,162,163,164]. The production of tyrosol, hydroxytyrosol, and tryptophol, originating from the catabolism of tyrosine and tryptophan, with hydroxytyrosol formed by hydroxylation of tyrosol, is also noteworthy. These compounds exhibit potent health-promoting activities, including antioxidant, anticarcinogenic, cardioprotective, and antimicrobial effects. For instance, tyrosol protect myocardial tissue against ischemia-induced stress [165] and to possess antimicrobial properties [166].

White wine has been associated with cardioprotective benefits due to the presence of tyrosol and caffeic acids, which activate cellular survival signaling pathways and the longevity-associated gene *FOXO3a*. Tyrosol also contributes to the sensory profile of alcoholic beverages, imparting bitterness at concentrations above the sensory threshold but below the recognition threshold [161]. In addition, yeast synthesizes glutathione (L-γ-glutamyl-L-cysteinyl-glycine, GSH), a significant antioxidant that scavenges reactive oxygen species (ROS), and trehalose, a disaccharide with notable bioactive functions [161]. GSH plays a central role in nutrient metabolism and the regulation of cellular processes, including DNA and protein synthesis, signal transduction, cell proliferation, and apoptosis. Trehalose exhibits anti-inflammatory properties and protects cellular membranes and labile proteins against denaturation caused by desiccation and oxidative stress [161].

Yeasts isolated from alcoholic beverages, namely wine, represent a promising alternative to probiotic bacteria due to their intrinsic antibiotic resistance. This property may help prevent antibiotic-associated intestinal disorders and mitigate the development of antibiotic resistance [167]. Consequently, research interest has expanded beyond *Saccharomyces boulardii* and *S. cerevisiae* to include other yeast species with potential probiotic properties. Several genera, including *Debaryomyces*, *Kluyveromyces*, *Yarrowia*, *Torulaspora*, *Candida*, *Pichia*, *Hanseniaspora*, *Metschnikowia*, and *Lachancea*, have been proposed as candidates with health-promoting potential [147]. However, to date, only *S. boulardii* has been formally recognized as a probiotic yeast; the remaining species require further in vitro and in vivo characterization before evaluation in human clinical trials [147,168].

As also highlighted by Todorov et al. [160], lactic acid bacteria play a pivotal role in both wine and vinegar production by synthesizing organic acids, melanoidins, and other bioactive compounds with antioxidant, anti-inflammatory, and antimicrobial properties [150,151,169]. In wine, lactic acid bacteria are responsible for the second fermentation process, known as malolactic fermentation, which involves the decarboxylation of L-malic acid to L-lactic acid. A wide range of lactic acid bacteria species are associated with malolactic fermentation including: (i) Facultative Heterofermentative bacilli (*Lactobacillus casei*, *Lb. coryniformis*, *Lb. curvatus*, *Lb. homohoichii*, *Lb. paracasei*, *Lb. pentosus*, *Lb. plantarum*, *Lb. sakei*, *Lb. zeae*, *Lb. nagelli*, *Lb. diolivorans*); (ii) Heterofermentative bacilli (*Lactobacillus brevis*, *Lb. buchnerii*, *Lb. collinoides*, *Lb. fermentum*, *Lb. fructivorans*, *Lb. hilgardii*, *Lb. kunkeei*, *Lb. sanfrancisensis*, *Lactobacillus* spp., *Lb. vacinostercus*); (iii) Homofermentative bacilli (*Lactobacillus delbrueckii*, *Lb. jensenii*, *Lb. mali*, *Lb. vini*); (iv) Homofermentative cocci (*Pediococcus acidilactici*, *Pc. damnosus*, *Pc. dextrinicus*, *Pc. inopinatus*, *Pc. parvulus*, *Pc. pentosaceus*, *Pediococcus* spp., *Lactococcus lactis*, *Lactococcus* spp., *Enterococcus* spp.) as well as heterofermentative cocci (*Leuconostoc citrovorum*, *Leuc. mesenteroides* subsp. *dextranicum*, *Leuc. mesenteroides* subsp. *mesenteroides*, *Leuconostoc* spp., *Weissella confusa*, *W. paramesenteroides*, *Weissella* spp., *W. uvarum*, *Oenococcus oeni* [147].

Wine therefore represents a rich reservoir of lactic acid bacteria, many of which exhibit probiotic potential. Several lactic acid bacteria species are already incorporated into commercial probiotic formulations, suggesting that less-explored strains originating from the wine microbiome may hold valuable functional properties. Table 6 summarizes the key bioactive compounds in wine and wine vinegar, their microbial sources, and their associated biological functions.

The use of microbial consortia composed of different yeast or bacterial strains can significantly enhance the production of specific bioactive compounds in both wine [161] and vinegar [136,151,163,164]. Bioaugmentation and co-culture fermentations strategies (e.g., involving lactic acid bacteria and ester-producing yeasts) have been shown to improve flavor, increase phenolic content, and enhance antioxidant and antimicrobial activities [151,169]. Moreover, the selection of raw material and microbial starter cultures plays a critical role in determining the final bioactive profile and sensory attributes of the fermented products [149,172,174].

## 5. Gut Microbiota Modulation by Bioactive Compounds

### 5.1. Gut Microbiome: Diversity, Dysbiosis, and Health Implications

The human gut microbiota comprises a highly diverse community of microorganisms, including bacteria, archaea, fungi, viruses, and protozoa, that inhabit the gastrointestinal tract, mainly the large intestine [36,175]. According to Craig [176] and Bäckhed [177], the dominant microbial phyla are *Bacteroidetes*, *Firmicutes*, *Actinobacteria*, *Proteobacteria*, *Fusobacteria*, and *Verrucomicrobia*, with *Firmicutes* and *Bacteroidetes* together accounting for more than 90% of the total microbial population. Through dynamic interactions with the host throughout life, these microorganisms exert a significant influence on overall health [36]. Recent advances in metagenomic sequencing, metabolomics, transcriptomics, and functional analyses have emphasized the vital role of the gut microbiota in maintaining homeostasis and regulating multiple physiological systems [37].

The establishment and development of the gut microbiome begin at birth, and its composition is strongly influenced by genetic, nutritional, and environmental factors, as well as age, lifestyle, and medication use. Changes in the composition and functional activity of the gut microbiota, commonly referred to as dysbiosis, can affect intestinal barrier integrity and disrupt digestive, metabolic, and immune functions. As described by Yonekura [178], the relationship between the gut microbiota and host health is bidirectional; pathological conditions can induce dysbiosis, while microbial imbalance, in turn, contributes to disease progression and worsening. Dysbiosis, often characterized by decreased microbial diversity, has been linked with a wide range of diseases [179,180]. Notable examples include inflammatory and metabolic disorders, such as obesity, type II diabetes mellitus, non-alcoholic fatty liver disease, cardiometabolic disorders, and malnutrition [181,182,183]. Furthermore, growing evidence suggests that alterations in intestinal microbial composition play a relevant role in carcinogenesis, especially in the development of colorectal cancer [184].

**Figure 3:**
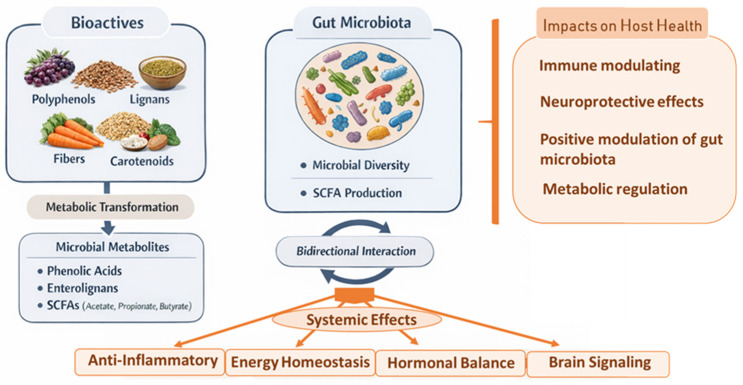
The relationship between gut microbiota and bioactive compounds and their impact on host health (Short-chain fatty acids—SCFAs).

In parallel, dysregulation of the gut–brain axis constitutes one of the mechanisms by which dysbiosis is implicated in neurological and neuropsychiatric diseases, including Alzheimer’s disease [185,186,187] and major depressive disorder [188,189]. However, defining an “optimal” health-promoting microbiota profile remains challenging, given the high interindividual variability, which is strongly influenced by environmental factors and intrinsic host characteristics [180]. In this context, bioactive compounds present in food emerge as natural modulators of the gut microbiota, with growing evidence supporting their potential role in disease prevention and management. Among these, phenolic compounds play a pivotal role in modulating the gut microbiota ecology by interacting with microbial communities, and modulating both composition and functionality (Figure 3).

### 5.2. Bioactive Compounds and Their Primary Sources

Bioactive compounds are food-derived molecules that, although not essential for survival, exert beneficial physiological effects due to their diverse biological properties [190,191,192,193]. They are widely distributed in fruits, vegetables, cereals, legumes, coffee, green tea, and animal-derived foods [37,194,195]. Regular consumption of bioactive compounds has been associated with favorable modulation of gut microbiota composition and regulation of relevant metabolic pathways involved in health maintenance and disease prevention [37,127].

Table 7 summarizes the relationships among gut microbiota taxonomy, dietary bioactive compounds, their primary health benefits, and their main food sources.

### 5.3. Mechanisms of Gut Microbiota Modulation by Bioactive Compounds

Bioactive compounds can act as prebiotics by selectively stimulating the growth of beneficial microorganisms [204]. Prebiotics intake promotes the enrichment of health-associated gut microorganisms, with a pronounced stimulatory effect on *Lactobacillus* and *Bifidobacterium* species [38]. De Cossío et al. [218] reported that inulin supplementation increases *Bifidobacterium* populations while simultaneously modulating the balance between Gram-positive and Gram-negative bacteria. Conversely, microbial fermentation of inulin and fructo-oligosaccharides generates short-chain fatty acids, predominantly acetate and butyrate. These metabolites lower intestinal pH inhibit pathogenic bacteria and activate specific G-protein-coupled receptors (GPCRs) involved in host energy metabolism and immune regulation [39,219].

Polyphenols represent a heterogeneous class of bioactive compounds, among which resveratrol and flavonoids are well known for their antioxidant and anti-inflammatory properties. Resveratrol, found in grapes, red wine, berries, and peanuts, has been shown to increase the abundance of *Lactobacillus* and *Bifidobacterium* species [199,200,205,206] and to modulate the Bacteroidetes-to-Firmicutes ratio in animal models [209]. Crucially, emerging evidence highlights potential adverse effects that warrant careful consideration when using non-flavonoid polyphenols. Resveratrol, for example, exhibits low bioavailability and can act as a pro-oxidant at high concentrations, potentially leading to cellular damage [220]. Furthermore, extensive microbial metabolism of polyphenols in the gut generates diverse metabolites, some of which may have uncertain or potentially harmful biological effects [221].

Dietary lignans, present in oilseeds are converted by the gut microbiota into entero-lignans, which are mediate many of their reported health benefits. However, evidence indicates that the antiestrogenic effects observed with high lignan intake are attributable to enterolignans rather than to the parent lignan compounds. Consequently, excessive exposure to enterolignans may contribute to disturbances in hormonal balance, in a manner comparable to that reported for resveratrol [222,223].

Phenolic acids, particularly hydroxybenzoic acids such as caffeic acid, ferulic acid, and *p*-coumaric acid, contribute to the maintenance of gut microbiota diversity and stability by promoting the growth of beneficial bacteria [224]. Dietary supplementation with caffeic acid has been shown to alter gut microbial composition by reducing *Bacteroides* and *Turicibacter*, while increasing *Alistipes* and *Dubosiella*. Similarly, Hu et al. [225] demonstrated that vanillic acid supplementation in piglets favorably modulates the gut microbiota by increasing the *Bacteroidetes/Firmicutes* ratio, decreasing *Prevotellaceae*, and rising populations of *Lachnospiraceae*, *Lachnospira*, *Eubacterium eligens*, and *Eubacterium*. These microbial shifts are associated with the reduced intestinal inflammation, supporting the role of phenolic acids in immunometabolic regulation.

The interaction between flavonoids and the gut microbiota is fundamental for regulating microbial community composition throughout the gastrointestinal tract and enhancing probiotics functionality. This bidirectional relationship also enhances the bioavailability and bioactivity of flavonoids, thereby strengthening their regulatory effects on intestinal disorders [204].

Bioactive compounds present in fruits, such as polysaccharides, dietary fiber, phenolic compounds, flavonoids, and carotenoids, play an essential role in modulating gut microbiota composition, diversity, and abundance [226]. These compounds interact with lactic acid bacteria, help rebalance the *Firmicutes*/*Bacteroides* ratio, and inhibit the growth of potentially pathogenic microorganisms [227]. Peerakietkhajorn et al. [228] demonstrated that specific oligosaccharides selectively alter microbial composition in both the proximal and distal colon by stimulating beneficial bacteria while reducing *Enterococcus* species, particularly *E. faecalis* and *E. faecium*, which are associated with adverse health effects.

Quercetin, a flavonoid widely distributed in fruits and vegetables, has gained considerable attention for its capacity to modulate the intestinal microbiota. Recent studies indicate that quercetin may exert prebiotic-like effects by increasing populations of beneficial bacteria such as *Bifidobacterium* and *Akkermansia*, while reducing potentially harmful intestinal microorganisms [207,210].

Regular consumption of fiber-rich diets modulates the intestinal microbiota by reducing Firmicutes abundance, increases the Bacteroidetes/Firmicutes ratio, and inhibits the proliferation of pathogenic microorganisms, thereby promoting a more balanced microbiota and improved intestinal health [37].

Fermentation of dietary fiber by intestinal bacteria produces short-chain fatty acids, primarily acetate, propionate, and butyrate [229], which are essential to maintaining intestinal barrier integrity and modulating immune responses, particularly in individuals with metabolic disorders such as obesity and diabetes. Short-chain fatty acids induced colonic acidification, thereby inhibiting harmful bacteria and favoring the growth of beneficial species. Increased dietary fiber intake can therefore remodel the intestinal microbiota by enriching populations of *Faecalibacterium prausnitzii* and other propionate-producing microorganisms [230,231], while reducing undesirable microorganisms such as *Escherichia coli* and *Pseudomonas* spp. [232].

Modulation of the gut microbiota by bioactive compounds represents a promising strategy for promoting health and preventing chronic diseases. Despite substantial progress, important knowledge gaps remain regarding specific mechanisms, interindividual variability, and the standardization of effective doses. Continued research is essential to translate these findings into practical nutritional strategies and clinical applications.

### 5.4. Dietary FODMAPs as Additional Modulators of the Gut Microbiota

In addition to bioactive compounds, other intrinsic food constituents exert a significant influence on intestinal microbial ecology. Among these, fermentable oligosaccharides, disaccharides, monosaccharides, and polyols (FODMAPs) constitute a group of short-chain carbohydrates commonly found in fruits, vegetables, cereals, legumes, dairy products, and various sweeteners [233,234]. Due to their limited absorption in the small intestine, FODMAPs reach the colon virtually intact, where they undergo rapid microbial fermentation, leading to increased gas production and the synthesis of short-chain fatty acids [39].

From a microbiological perspective, regular consumption of FODMAP containing foods may support saccharolytic fermentation and stimulate the growth of beneficial bacteria, such as *Bifidobacterium*, thereby contributing to the production of short-chain fatty acids and mucosal homeostasis [235]. However, in individuals with visceral hypersensitivity, altered gastrointestinal motility, or reduced absorptive capacity, this rapid fermentation may exacerbate gastrointestinal symptoms, including bloating, abdominal distension, abdominal pain, and altered bowel movements. These characteristics are commonly associated with irritable bowel syndrome (IBS) [236].

Clinical trials and meta-analyses consistently demonstrate that reducing fermentative substrates via a low-FODMAP diet decreases luminal gas formation and osmotic load, resulting in significant symptom improvement in a substantial proportion of individuals with IBS [236,237,238]. Due to its relatively simple implementation, a low-FODMAP dietary pattern can be beneficial and well-tolerated by a large portion of the population with functional gastrointestinal complaints.

However, FODMAP restriction is not without limitations. Evidence suggests that prolonged adherence to a strict low-FODMAP diet may reduce microbial diversity and decrease the abundance of health-promoting taxa, particularly *Bifidobacterium* spp. [42,239]. For this reason, current dietary guidelines recommend a structured approach, comprising an initial restriction phase, followed by gradual reintroduction and personalized adjustment of FODMAP intake. This strategy aims to balance symptom management with the maintenance of long-term microbial stability and diversity [41,240,241].

## 6. Mechanisms of Action in Human Health

Food-derived bioactive compounds exert pleiotropic health effects by modulating a limited number of interconnected molecular pathways rather than acting on isolated physiological systems. Central to these effects are the regulation of redox homeostasis, in-flammatory signaling, and cellular energy metabolism, which together influence gene ex-pression, metabolic flexibility, and tissue function. This shared mechanistic framework provides a biological basis for the protective effects attributed to polyphenols, carotenoids, omega-3 fatty acids, and other phytochemicals, and underpins their potential role in the prevention and management of chronic diseases.

### 6.1. Anti-Inflammatory and Redox Mechanisms

Several bioactive compounds modulate inflammation processes by targeting intracellular signaling cascades. Polyphenols and flavonoids, for instance, downregulate the nuclear factor kappa B (NF-κB) pathway, thereby reducing the expression of pro-inflammatory cytokines such as interleukin-6 (IL-6), tumor necrosis factor-alpha (TNF-α), and interleukin1β (IL-1β). In parallel, carotenoids and curcumin inhibit cyclooxygenase-2 (COX-2) activity and eicosanoid synthesis, attenuating the chronic low-grade inflammation that underlies many non-communicable diseases. In addition, several bioactive compounds suppress activation of the NOD-like receptor family pyrin domain containing 3 (NLRP3) inflammasome, a mechanism implicated in metabolic, autoimmune, and neurological disorders; however, the majority of supporting evidence remains confined to cell culture models underscoring the need for robust translational research to establish clinical relevance [242].

Redox regulation represents a complementary mechanism underlying these anti-inflammmatory effects. Polyphenols and carotenoids can directly scavenger reactive oxygen and nitrogen species (ROS/RNS) and activate endogenous antioxidant defenses via the Nrf2/ARE signaling pathway, leading to the upregulation of enzymes such as superoxide dismutase, catalase, and glutathione peroxidase. Together, these mechanisms highlight the close interplay between oxidative stress and inflammation in mediating the health effects of bioactive compounds.

### 6.2. Metabolic Regulation and Anti-Diabetic Effects

Bioactive compounds further modulate cellular energy metabolism through key regulatory pathways. Activation of AMP-activated protein kinase (AMPK) enhances fatty acid oxidation and glucose uptake, thereby improving metabolic flexibility and insulin sensitivity. Concurrent inhibition of the mechanistic target of rapamycin (mTOR) pathway promotes autophagy and cellular repair processes, with important implications for aging and metabolic disorders. Collectively, these mechanisms highlight the role of dietary bioactives in maintaining energy homeostasis and metabolic health.

Evidence from animal models and emerging clinical studies supports the antidiabetic potential of fenugreek-derived bioactives, such as soluble dietary fibers, 4-hydroxyisoleucine, flavonoid glycosides, saponins, and trigonelline. These compounds exert their effects through multiple mechanisms, including delayed gastric emptying, reduced intestinal glucose absorption, improved insulin secretion, and modulation of the gut microbiota [243].

Clinical trials have further demonstrated that algae-derived extracts can regulate blood glucose levels and alleviate diabetes-related complications [244]. Evidence synthetized from intervention studies and summarized in recent systematic reviews and meta-analyses indicates that, in individuals with type 2 diabetes mellitus (T2DM), supplementation with black cumin, cinnamon, and ginger results in significant reductions in fasting glucose (−27 to −17 mg/dL). Among these interventions, ginger and black cumin were associated with improvements in glycated hemoglobin (HbA1c), while cinnamon and ginger decreased insulin levels. These effects appear to be mediated by inhibition of α-amylase and α-glucosidase, attenuation of oxidative stress, preservation of pancreatic β-cell integrity, and enhanced insulin sensitivity [245].

Similarly, glucosinolates derived from broccoli sprouts have been shown to reduce serum insulin levels and improve insulin resistance in patients with T2DM [246]. Tomato consumption, which provides lycopene, catechins, and phenolic acids, has also been associated with improved glycemic control, potentially via α-glucosidase inhibition, activation of the phosphoinositide 3-kinase (PI3K)-eNOS/NO pathway, and enhanced insulin sensitivity [247].

Metabolic syndrome, characterized by hyperglycemia, obesity, hypertension, hypertriglyceridemia, and low HDL cholesterol, is one of the most prevalent global health conditions and is strongly linked to insulin resistance. Several clinical studies report that green coffee consumption reduces risk factors associated with metabolic syndrome by improving blood pressure, lipid profiles, and body weight [248]. This condition is also associated with a systemic pro-inflammatory state that increases the risk of T2DM and cardiovascular diseases.

Fruits and vegetables rich in antioxidant and anti-inflammatory phytochemicals, have been shown in meta-analyses of clinical trials to significantly reduce C-reactive protein (CRP) levels, particularly anthocyanins (*p* = 0.001) and plant oils (*p* < 0.00001) [249]. In vivo studies further suggest that plant essential oils (PEOs), composed of complex mixtures of volatile and lipophilic compounds, inhibit multiple dysregulated inflammatory pathways, including Toll-like receptors, NF-κB, MAPKs, and NLRP3 inflammasome activation, as well as antioxidant pathways such as Nrf2/ARE and JAK/STAT signaling. Nevertheless, clinical evidence supporting the anti-inflammatory efficacy of PEOs remains limited underscoring the need for well-designed human intervention studies [250,251].

### 6.3. Cardioprotective Mechanisms

The cardioprotective effects of bioactive compounds are primarily mediated through improvements in vascular function and lipid metabolism. Flavonoids and nitrate-rich vegetables enhance endothelial nitric oxide bioavailability, thereby promoting endothelium-dependent vasodilation and lowering blood pressure. Polyphenols inhibit low-density lipoprotein (LDL) oxidation and platelet aggregation, reducing the risk of atherosclerosis and thrombotic events. Substantial clinical and experimental evidence supports the cardioprotective effects of omega-3 fatty acids, particularly docosahexaenoic acid (DHA) and eicosapentaenoic acid (EPA), which exert complementary actions through membrane incorporation and regulation of blood lipids, blood pressure, and atherosclerosis processes [252]. Olive oil, rich in phenolic compounds, has demonstrated reductions in LDL-cholesterol, apolipoprotein B, and the LDL/HDL ratio in vivo [253]. At the same time, epidemiological studies associate olive oil consumption with reduced cardiovascular (16%) and cancer (11%) mortality [254].

Blueberries, which are rich in anthocyanins, improve markers of vascular function, including flow-mediated dilation (FMD) and reactive hyperemia index (RHI), primarily through activation of the eNOS/NO/cGMP signaling pathway [255]. Chocolate, another food rich in flavonoids, has been inversely associated with cardiovascular and cancer mortality when consumed as part of calorie-balanced diets [256]. Other flavonoids such as anthocyanins, hesperidin, quercetin, and epigallocatechin gallate (EGCG), modulate lipid profiles, partly by inhibiting cholesteryl ester transfer protein (CETP), thereby increasing HDL cholesterol, and reducing triglycerides and total cholesterol levels [257]. Garlic-derived bioactives, including organosulfur compounds, saponins, and quercetin, have been shown to lower blood pressure, LDL-cholesterol, and triglyceride levels, reduce inflammatory markers, and improve vascular parameters such as carotid intima-media thickness [258]. Tomato bioactives, including lycopene, quercetin, and vitamin C, further contribute to reductions in blood pressure and LDL oxidation through mechanisms involving enhanced nitric oxide bioavailability, inhibition of NADPH oxidase, and decreased platelet aggregation [247].

### 6.4. Epigenetic Regulation and Antitumor Activity

Bioactive compounds also exert pleiotropic effects through antioxidant and epigenetic mechanisms. Polyphenols and carotenoids can directly scavenger reactive oxygen and nitrogen species (ROS/RNS) and activate endogenous antioxidant defenses via the Nrf2/ARE signaling pathway, leading to the upregulating of enzymes such as superoxide dismutase, catalase, and glutathione peroxidase.

Epigenetic regulation by bioactive compounds such as resveratrol, epigallocatechin gallate, and curcumin involves modulation of DNA methylation, histone modifications, and microRNA regulation, with important implications for carcinogenesis, metabolism, and aging. Algal biomass-derived bioactives are currently under evaluation in clinical trials for their anticancer properties, showing inhibitory effects against breast, myeloma, and prostate cancer cells [244].

Bioactives derived from the *Brassicaceae* family, particularly isothiocyanates such as sulforaphane, exhibit significant antitumor activity against colon [259] and prostate cancers, with evidence suggesting delayed biochemical recurrence in prostate cancer patients [246,260]. Additionally, phenolic compounds from extra-virgin olive oil have been associated with epigenetic modifications, although the underlying causal mechanisms have yet to be fully elucidated [261].

### 6.5. Immunomodulation and Neuroprotection

Bioactive compounds further contribute to immunomodulation. Polyphenols enhance the activity of natural killer (NK) cells and macrophages and promote the differentiation of regulatory T cell, thereby limiting excessive inflammatory responses [262]. Algae-derived extracts have demonstrated immunomodulatory effects in humans, potentially enhancing antibody production and improving host resistance to viral infections [244]. Bioactives derived from kefir-derived bioactives, including exopolysaccharides and peptides, exhibit antimicrobial, anticancer, and immunomodulatory properties through multiple signaling pathways [263].

Neuroprotective effects of bioactives are mediated through antioxidant, anti-inflammatory, and microbiota-dependent mechanisms. Flavonoids and omega-3 fatty acids support synaptic plasticity and neuronal survival by modulating pathways involved in memory and learning. Short-chain fatty acids produced by the gut microbiota contribute to gut–brain axis signaling and may provide therapeutic benefits in neurodegenerative disorders. Clinical investigations include brown algae-derived amino acids currently in phase II trials for Alzheimer’s disease [244]. Anthocyanins from blueberry have been associated with improved cognitive performance, including significant benefits for working memory [264]. In vivo and in vitro studies further suggest that flavonoids, alkaloids, and terpenoids exert anticonvulsant activity by modulating γ-aminobutyric acid (GABA), N-methyl-D-aspartate (NMDA), and cannabinoid receptors, as well as ion channels, in addition to their antioxidant actions [234]. Tomato -derived carotenoids, particularly lutein and zeaxanthin contribute to ocular health by protecting against age-related macular degeneration through singlet oxygen quenching and retinal protection [247].

### 6.6. Processing, Bioavailability, and Translational Relevance

Table 8 provides a concise overview of the principal mechanisms through which bioactive compounds influence human health. It highlights key molecular targets, representative compounds, and their associated physiological outcomes. This comparative summary illustrates how different classes of bioactives converge on common signaling pathways, while exerting distinct effects relevant to chronic disease prevention and overall health promotion.

**Figure 4:**
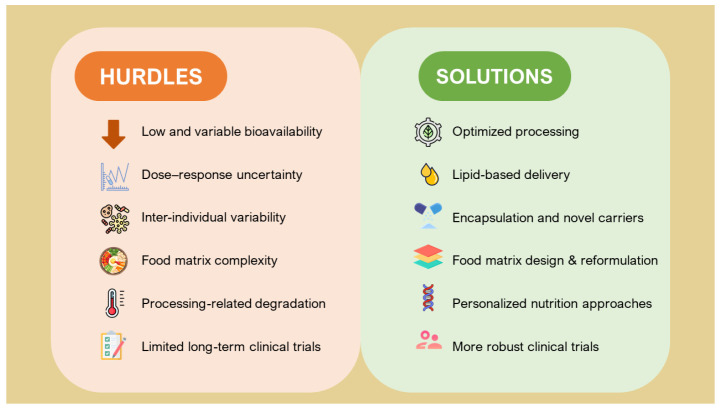
Overview of the main hurdles limiting the translation of bioactive compound mechanisms into measurable health outcomes, along with potential strategies to enhance their stability, bioavailability, and clinical efficacy.

It should be noted that the bioavailability of food-derived bioactive compounds is a complex, multistep process involving liberation from the food matrix, absorption, distribution, metabolism, and elimination. This process is strongly influenced by bioaccessibility, food matrix effect, transporter activity, molecular structures, metabolizing enzymes, and food processing practices, all of which can either enhance or impair absorption and physiological activity. Thermal treatments, mechanical homogenization, and fermentation can promote the release of carotenoids, polyphenols, and other bioactives from the food matrix, thereby improving micellarization and intestinal uptake. In addition, co-ingestion with dietary lipids markedly enhances the bioavailability of lipophilic compounds such as lycopene and lutein. In contrast, excessive oxidation, prolonged heating, or light exposure may compromise bioactive stability [24]. Consideration of these processing- and matrix-related factors is therefore essential for translating mechanistic insights into practical and evidence-based dietary recommendations (Figure 4).

## 7. Conclusions and Future Perspectives

Dietary intake is the primary source of antioxidants and a key determinant of the metabolic activities of the human gut microbiota. Among these antioxidants, polyphenols have received considerable attention due to their broad spectrum of biological activities, including anti-inflammatory, antioxidant, anticancer, and antidiabetic effects. Significant advances have been made in understanding the metabolism, bioaccessibility, and physiological functions of food-derived bioactive compounds. Although several challenges still hinder a comprehensive assessment of their health-promoting potential. One major constraint is the generally low bioavailability of many bioactives, which has stimulated the development of delivery and encapsulation strategies based on safe, edible materials to enhance stability, absorption, and efficacy; nevertheless, these approaches still present significant limitations [266,267].

Despite these challenges, the ability of dietary bioactives to modulate the human gut microbiota is increasingly recognized as a crucial mechanism underlying their health effects. The gut microbiota plays a central role in inflammation and metabolism, and growing evidence indicates that food bioactives influence microbial composition and function. However, the complex interactions among bioactives, gut microbiota, microbial-derived metabolites, and host metabolic and inflammatory pathways, particularly in the context of low-grade inflammation and metabolic syndrome remain incompletely understood.

Current evidence indicates that food-derived bioactive compounds exert pleiotropic effects through multiple, interconnected molecular pathways, including the regulation of oxidative stress, inflammation, epigenetic mechanisms, energy metabolism, cardiovascular function, immune responses, and neuroprotection. While these findings provide a strong mechanistic basis for their role in disease prevention and health promotion. Nevertheless, essential limitations remain in the existing literature. These include the short duration of many clinical trials, heterogeneous or suboptimal dosing strategies, and limited assessment of long-term safety and potential adverse effects. Consequently, well-designed, adequately powered, and longer-term human intervention studies are required to establish causal relationships and clarify clinical relevance.

Future research should prioritize elucidating the molecular mechanisms underlying interactions between specific phytochemicals and intestinal microorganisms; identifying how microbial taxa transform these compounds into bioactive or inactive metabolites, and determining how these transformations influence host physiology. Equally important is the characterization of gut microbiota and metabolite profiles associated with health and disease states, enabling the identification of reliable microbial and metabolic biomarkers. Studies should also address synergistic or antagonistic effects among co-consumed bioactive compounds and other dietary components, reflecting realistic dietary patterns rather than isolated exposures.

Additional research is needed to clarify the influence of the food matrix and processing conditions on bioaccessibility; microbial metabolism, and biological efficacy, as well as to account for inter-individual variability in gut microbiota composition and metabolic function. The application of integrated multi-omics approaches, including metagenomics, metabolomics, transcriptomics, and proteomics, will be essential to link dietary bioactives, microbial function, and host metabolic and inflammatory responses.

Finally, as bioactive compounds are primarily consumed through the diet, efforts must be directed toward overcoming challenges related to compound variability, standardization, bioavailability, and clinical validation. This includes the development of standardized bioactive preparations, harmonized analytical methodologies, validated endpoints, and rigorously designed human intervention trials. Addressing these gaps will be essential to the effective integration of food derived bioactives, particularly those from plant-based and fermented foods, into evidence-based public health strategies, functional food development, and personalized nutrition approaches aimed at the prevention and management of chronic diseases.

These efforts will ultimately support the development of functional foods, personalized nutrition strategies, and evidence-based interventions for the prevention and management of chronic disease.

## Figures and Tables

**Table 1:** Major classes of bioactive and phytochemical compounds, representative examples, and their key food sources.

Class of Bioactive/Phytochemical	Representative Examples	Key Foods	References
Flavonoids	Flavonols, Flavanols, Flavanones, Anthocyanins	Berries, onions, apples, citrus fruits	[42]
Phenolic Acids	Hydroxycinnamic acids, Hydroxybenzoic acids	Coffee, grapes, cherries, whole grains	[43]
Carotenoids	Beta-carotene, Lycopene, Lutein, Zeaxanthin	Carrots, tomatoes, spinach, kale	[44]
Glucosinolates/Isothiocyanates	Sulforaphane, Indole-3-carbinol	Broccoli, cauliflower, kale, Brussels sprouts	[45]
Alkaloids	Solanine, Tomatine, Capsaicin	Potatoes, tomatoes, peppers	[46]
Vitamins (bioactive micronutrients)	Vitamin C, Vitamin E, Folate	Citrus fruits, bell peppers, leafy greens	[47]
Terpenoids	Limonene, Lycopene	Citrus fruits, tomatoes	[48,49]
Probiotics/Fermented-food bioactives	Lactic acid bacteria, bioactive peptides	Yogurt, kimchi, sauerkraut	[50]

**Table 2:** Bioactive compounds and their commonly associated health effects.

Bioactive Compounds	Main Biological Effects	Reference
Phenolics, Anthocyanins	Antioxidant, anti-inflammatory, cardioprotective	[20]
Lutein, Zeaxanthin	Eye health, antioxidant, anti-inflammatory	[51]
Glucosinolates, Isothiocyanates	Anticancer, detoxification, anti-inflammatory	[52]
Vitamin C, Flavanones	Antioxidant, immune support, anti-inflammatory	[53]
Allicin, Quercetin	Cardioprotective, antimicrobial, anti-inflammatory	[54,55]
Lycopene, Beta-carotene	Antioxidant, cardioprotective, anticancer	[56,57]
Polyacetylenes	Antioxidant, anti-inflammatory, antiviral	[58]

**Table 5:** Examples of microbial diversity and bioactivity across major fermented food groups.

Fermented Food Type	Dominant Microbes/Diversity	Notable Bioactive Activities	References
Dairy (e.g., yogurt, cheese, kefir)	Lactic acid bacteria (*Lactobacillus*, *Streptococcus*, *Lactococcus*), *Acetobacter*	Probiotic effects, vitamin synthesis, antimicrobial peptides	[131,140,141,142]
Plant-based (e.g., kimchi, sauerkraut, fermented vegetables)	*Lactiplantibacillus plantarum*, *Leuconostoc*, *Lactobacillus fermentum*, *Enterobacteriaceae*	Antioxidant activity, polyphenol transformation, and antimicrobial compounds	[123,125,128,140]
Cereal-based (e.g., ogi, fufu, sourdough)	*Lactobacillus*, *Bacillus*, *Zymomonas*, yeasts	Vitamin B-group production, improved digestibility, and antimicrobial activity	[143,144,145]
Legume-based (e.g., fermented soy, locust bean)	*Bacillus*, *Lactobacillus*, *Staphylococcus*	Protein hydrolysis, bioactive peptides, and potential probiotic effects	[144,145,146]
Fermented beverages (e.g., kombucha, kvass, water kefir, wine, vinegar)	*Acetobacter*, *Gluconobacter*, *yeasts*, *Lactobacillus*, *Saccharomyces*, and *non-Saccharomyces* yeasts.	Antioxidant, antimicrobial, and potential probiotic properties	[131,136,140,145,147]

**Table 6:** Summary of key bioactive compounds, their microbial sources, and functions in wine and wine vinegar.

Compound Type	Main Microbial Source	Key Functions/Benefits	References
Indole metabolites	Yeasts (wine)	Antioxidant, potential neuroactive	[163,164,170]
Polyphenols/flavonoids	Yeasts, bacteria (both)	Antioxidant, antimicrobial	[149,171,172]
Melanoidins	LAB, acetic acid bacteria	Gut microbiota modulation, antioxidant	[150,173]
Organic acids	Acetic acid bacteria, LAB	Antimicrobial, flavor, health effects	[135,150,152,169,172,174]
Statin-like compounds	Acetic acid bacteria	Cholesterol-lowering potential	[149]

**Table 7:** Relationship between gut microbiota taxonomy and bioactive compounds associated with main health benefits.

Bioactive Compound/Subclass	Targeted Microbiota	Main Health Benefits	Sources	Reference
Polyphenols (general)	*Biofidobacteria*, *Lactobacilli*, *Clostridia* *Bifidobacterium* and *Lactobacillus* *Faecalibacterium prausnitzii* *Roseburia* species	Antioxidant and anti-inflammatory effects; positive modulation of gut microbiota	Green tea, blueberries, strawberries, raspberries, cocoa, red wine, apples, onions, curcumin (turmeric)	[196,197,198,199,200,201]
Flavonoids (polyphenol subclass)	*Bifidobacterium* spp. and *Lactobacillus* spp.	Antioxidant, anti-inflammatory, and prebiotic effects ↑ *Bifidobacterium* spp. and *Lactobacillus* spp.; ↓ pathogen *Clostridium perfringens*	Lemons, oranges, soybeans, parsley, *Ginkgo biloba*, Almonds	[196,202,203,204]
Anthocyanins (flavonoid subclass)		Physiological and functional benefits for health maintenance	Plant tissues, cranberries, raspberries, strawberries, red grapes	[196]
Resveratrol (specific polyphenol)	*Lactobacillus* and *Bifidobacterium*, *Firmicutes* and *Proteobacteria*	Modulates gut microbiota; ↑ *Lactobacillus* and *Bifidobacterium;* ↑ *Firmicutes/Proteobacteria*;	Grapes, red wine, berries	[205]
Quercetin (specific flavonoid)	*Bifidobacterium* and *Akkermansia*	Prebiotic effect: ↑ *Bifidobacterium* and *Akkermansia*; ↓ harmful gut bacteria	Various fruits and vegetables (apples, onions, citrus fruits)	[206,207,208,209,210]
Carotenoids		Antioxidant properties; ↓ inflammation, oxidative stress, and metabolic disorders	Colored fruits and vegetables (carrots, tomatoes)	[197,198]
Theanine (alkaloid)	*Lactobacillus* and *Bifidobacterium*	↑ *Lactobacillus* and *Bifidobacterium*	Green tea	[200]
Terpenes and Terpenoids		Anticancer, antimicrobial, antioxidant, antiallergic, and anti-inflammatory properties	Essential oils from plants such as oregano, thyme, lavender, citrus peel, Echinacea, and ginseng	[211]
Dietary fibers (e.g., inulin, oligosaccharides, resistant starch)	*Bifidobacterium*, *Lactobacillus*, and *Faecalibacterium prausnitzii*	Prebiotic effect: ↑ *Bifidobacterium*, *Lactobacillus*, and *Faecalibacterium prausnitzii*; ↑ short-chain fatty acids, regulate inflammation, support gut barrier integrity	Fiber-rich plant foods (grains, legumes, fruits, tubers)	[212,213,214,215,216]
Sulfur-containing compounds (e.g., allicin)		Antimicrobial effects; support beneficial gut bacteria; generation of bioactive metabolites in the gut	Garlic, onions, cruciferous vegetables (broccoli, cauliflower, kale)	[217]

Legend: ↑— increase, ↓—decrease.

**Table 8:** Mechanisms of action of food bioactive compounds, examples, and health outcomes.

Mechanism	Key Pathways/Targets	Examples of Bioactives	Main Health Outcomes	References
Antioxidant	Nrf2/ARE pathway; ↑ SOD, catalase, GPx; scavenging of ROS/RNS	Polyphenols (EGCG, resveratrol, quercetin), carotenoids (lycopene, β-carotene), vitamin C	Reduced oxidative stress; protection of DNA, proteins, and lipids	[247,252,253]
Anti-inflammatory	NF-κB downregulation; COX-2 inhibition; ↓ IL-6, TNF-α, IL-1β; inhibition of NLRP3 inflammasome	Curcumin, quercetin, lycopene, carotenoids, plant essential oils (PEOs)	Attenuation of chronic inflammation; reduced risk of non-communicable diseases	[242,245,250,251]
Epigenetic modulation	DNA methylation, histone acetylation, microRNA regulation	Resveratrol, EGCG, curcumin, sulforaphane (*Brassicaceae*), olive oil phenolics	Cancer prevention; modulation of aging and metabolism	[246,260,261]
Metabolic regulation	AMPK activation; mTOR inhibition; PI3K-eNOS/NO pathway; inhibition of α-amylase and α-glucosidase	Fenugreek bioactives (fibers, 4-HIL, diosgenin, trigonelline), black cumin, cinnamon, ginger, glucosinolates, lycopene, catechins, green coffee	Improved insulin sensitivity; enhanced glucose control; reduced metabolic syndrome risk	[243,244,245,246,247,248]
Cardiovascular protection	↑ NO bioavailability; ↓ LDL oxidation; platelet aggregation inhibition; CETP modulation	Flavonoids (anthocyanins, hesperidin, quercetin, EGCG), nitrate-rich vegetables, omega-3 fatty acids (EPA, DHA), olive oil phenolics, garlic compounds, tomato bioactives	Reduced blood pressure, atherosclerosis, and thrombosis; improved lipid profile; lower cardiovascular mortality	[247,252,253,254,255,256,257,258]
Immunomodulation	NK cell activation; macrophage activity; Treg differentiation; antibody production	Polyphenols, β-glucans, carotenoids, algae extracts, kefir bioactives (kefiran, peptides)	Stronger immune defense; improved response to infection; reduced autoimmunity	[244,262,263]
Neuroprotection	Synaptic plasticity; antioxidant and anti-inflammatory effects; modulation of gut–brain axis; neurotransmitter receptor targets (GABA, NMDA)	Flavonoids, omega-3 fatty acids, blueberry anthocyanins, algae amino acids, terpenoids, alkaloids	Cognitive support; improved memory and learning; prevention of neurodegeneration and seizures	[244,264,265]
Ocular health	Singlet oxygen quenching; retinal protection	Lutein, zeaxanthin (from tomatoes and other vegetables)	Reduced risk of age-related macular degeneration (AMD)	[247]

Legend: ↑—increase, ↓—decrease.

## Data Availability

No new data were created or analyzed in this study. Data sharing is not applicable to this article.
